# Supplementary material for: Drug repurposing for aging research using model organisms
Source: Aging Cell. 2017 Jun 16;16(5):1006–15. doi: 10.1111/acel.12626 (PMC5595691; doi:10.1111/acel.12626)
Supplement: Supplementary file 7 — Data S1 Zip‐Archive of all report cards. [file ACEL-16-1006-s007.zip › RC_IQK.pdf]

IQK

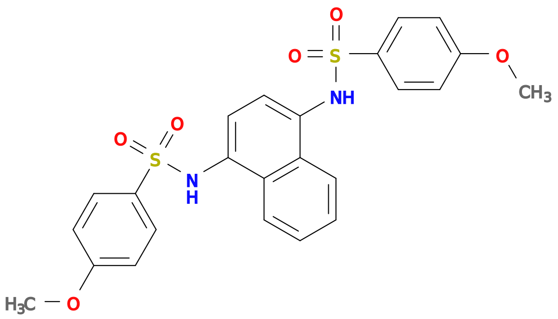

Database identifiers

ChEMBLCompound	CHEMBL2402207
ZINC	ZINC00781579
eMolecules	1370355

Ranking

	Rank	Score
Drosophila	472/697	0.302
C. elegans	NA	NA

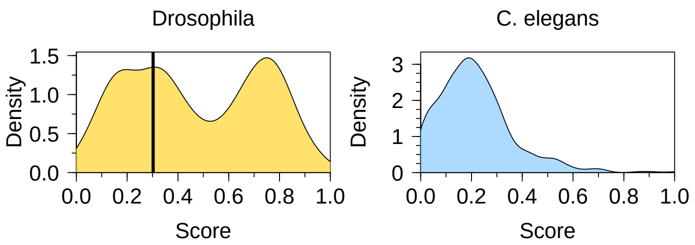

	Ageing implication	Domain conservation	Binding site conservation	Binding affinity	Bioavailability	Lipinski	Promiscuity	Purchasability	Drug approval	**Total**
Drosophila	0.36	0.929	0.956	0.878	(0.9)	-0.05	-0.0	0.1	0.0	0.302
C. elegans	NA	NA	NA	NA	NA	NA	NA	NA	NA	NA

Names

No synonyms found

Roles

ChEBI entry None has no roles

Status

Approved drug (according to ChEMBL)	No
Number of Rule of 5 violations	1
Binding affinity to original target in log units (RF-Score prediction)	6.98
Burns *C. elegans* bioavailability prediction	-8.62

Compound Target Characteristics

Kelch-like ECH-associated protein 1

Best gene implication in ageing for this target family came from gene Q8IGL4 via mapping the annotation from Ensembl FBgn0038475 via mapping the annotation from EntrezGene 42062 via mapping the annotation from GenAgeModels 0859 annotated in GenAge release 17.

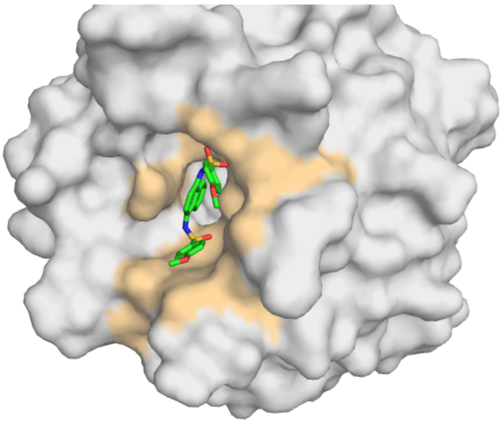

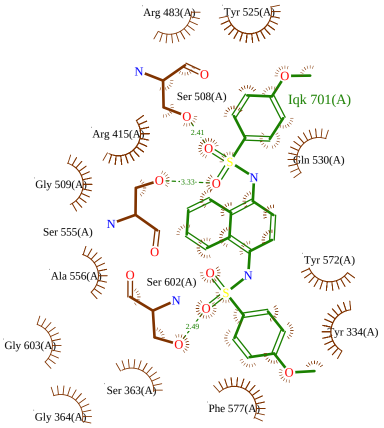

protein	amino acids contacts (binding site)														
PDB:4iqk:chainA:Q14145	Y	S	G	R	R	S	G	Y	Q	S	A	Y	F	S	G
sp:Q14145:KEAP1_HUMAN	Y	S	G	R	R	S	G	Y	Q	S	A	Y	F	S	G
tr:G3V8U2:G3V8U2_RAT	Y	S	G	R	R	S	G	Y	Q	S	A	Y	F	S	G
tr:Q3UWI4:Q3UWI4_MOUSE	Y	S	G	R	R	S	G	Y	Q	S	A	Y	F	S	G
sp:Q9Z2X8:KEAP1_MOUSE	Y	S	G	R	R	S	G	Y	Q	S	A	Y	F	S	G
tr:Q8IGL4:Q8IGL4_DROME	F	S	G	R	R	S	G	F	Q	S	A	F	F	S	G
tr:Q9VEN5:Q9VEN5_DROME	F	S	G	R	R	S	G	F	Q	S	A	F	F	S	G
tr:A9UNI1:A9UNI1_DROME	F	S	G	R	R	S	G	F	Q	S	A	F	F	S	G
tr:Q7KSF5:Q7KSF5_DROME	F	S	G	R	R	S	G	F	Q	S	A	F	F	S	G

	whole protein		domain-based		contact-based	
protein	ident	simil	ident	simil	ident	simil
PDB:4iqk:chainA:Q14145	0.99	1.0	0.99	1.0	1.0	1.0
sp:Q14145:KEAP1_HUMAN	1.0	1.0	1.0	1.0	1.0	1.0
tr:G3V8U2:G3V8U2_RAT	0.94	0.98	0.96	0.99	1.0	1.0
tr:Q3UWI4:Q3UWI4_MOUSE	0.6	0.62	0.97	0.99	1.0	1.0
sp:Q9Z2X8:KEAP1_MOUSE	0.94	0.98	0.97	0.99	1.0	1.0
tr:Q8IGL4:Q8IGL4_DROME	0.31	0.5	0.54	0.86	0.8	0.96
tr:Q9VEN5:Q9VEN5_DROME	0.37	0.63	0.54	0.86	0.8	0.96
tr:A9UNI1:A9UNI1_DROME	0.36	0.63	0.54	0.86	0.8	0.96
tr:Q7KSF5:Q7KSF5_DROME	0.36	0.63	0.54	0.86	0.8	0.96

Keap1 (FBgn0038475) associated phenotypes

developmental rate defective, dominant, large body, long lived, male limited, oxidative stress response defective

(Information from FlyBase)
